# Multiplexed Spectral Imaging of 120 Different Fluorescent Labels

**DOI:** 10.1371/journal.pone.0158495

**Published:** 2016-07-08

**Authors:** Alex M. Valm, Rudolf Oldenbourg, Gary G. Borisy

**Affiliations:** 1 Marine Biological Laboratory, Woods Hole, Massachusetts, United States of America; 2 Brown University, Providence, Rhode Island, United States of America; 3 Forsyth Institute, Cambridge, Massachusetts, United States of America; University of California Irvine, UNITED STATES

## Abstract

The number of fluorescent labels that can unambiguously be distinguished in a single image when acquired through band pass filters is severely limited by the spectral overlap of available fluorophores. The recent development of spectral microscopy and the application of linear unmixing algorithms to spectrally recorded image data have allowed simultaneous imaging of fluorophores with highly overlapping spectra. However, the number of distinguishable fluorophores is still limited by the unavoidable decrease in signal to noise ratio when fluorescence signals are fractionated over multiple wavelength bins. Here we present a spectral image analysis algorithm to greatly expand the number of distinguishable objects labeled with binary combinations of fluorophores. Our algorithm utilizes a priori knowledge about labeled specimens and imposes a binary label constraint on the unmixing solution. We have applied our labeling and analysis strategy to identify microbes labeled by fluorescence in situ hybridization and here demonstrate the ability to distinguish 120 differently labeled microbes in a single image.

## Introduction

Many biological structures are made up of numerous discrete substructures, from the many different species of microbes that comprise biofilms to the multiple organelles and macromolecular assemblies that make up the eukaryotic cell [1–3]. Molecular based approaches such as genomic sequencing, mRNA expression profiling, and proteomic analyses provide exhaustive information on the components of complex biological systems from which meaningful biological relationships can be inferred [4]. However, deciphering interrelationships is limited in part because these molecular based approaches give very little information on the spatial arrangement of the system components on the scale at which these structures are known to interact.

Fluorescence imaging has proven to be an exquisitely sensitive method for analyzing the spatial structure of biological organization on scales from tens of nanometers to hundreds of microns. However, with few notable exceptions [5–8] a general method of systems-level fluorescence imaging has not been achieved. In principle, many different kinds of individual substructures, be they molecular complexes, organelles or microbial cells, could be labeled with unique fluorescent reporters, as the number of small organic fluors and fluorescent proteins is already substantial and ever increasing [9,10]. Such information would be highly beneficial in deconstructing and understanding organization at many levels within biological systems. However, in practice, the number of different moieties distinguishable in a single image by fluorescence microscopy is severely limited by the spectral overlap of their fluorophore labels when imaged through bandpass filters [11].

One method that has been used previously to increase the number of distinguishable objects in fluorescence imaging is combinatorial labeling wherein biological targets are labeled with specific combinations of fluorophore reporters. This approach has been used to successfully distinguish multiple different axonal processes in transgenic mice expressing stochastic combinations of three fluorescent proteins as well as to distinguish up to 32 different mRNA transcripts in yeast [3,5,8]. Both approaches use a priori information about the spatial structure of the labeled specimens, either that individual cells expressing specific combinations of fluorescent labels cannot co-localize in space or that the spacing of the fluorophore labels on an approximately linear mRNA molecule is known.

A second approach that has been used to distinguish multiple fluorescently labeled objects in biological images is spectral imaging and subsequent linear unmixing of spectrally recorded data. This approach has been used to distinguish multiple markers in cells [12]. With spectral imaging and linear unmixing there is no requirement that fluorescent labels be segmented in space as the unmixing algorithm finds the best linear fit of any combination of fluorescent spectra at every pixel in an image.

Spectral imaging coupled with linear unmixing overcomes the limitations imposed by spectral overlap and bandpass filters [13,14]. However, the linear unmixing algorithm has itself a fundamental limit to the number of labels it can distinguish. Because the algorithm assigns fluorophore identity to measured light intensity by solving a set of linear equations, the maximum number of different labels that can be unambiguously identified in an image is limited by the number of spectral channels used to record the digital image. Further, the confidence in label assignment relies heavily on the signal to noise ratio (SNR) in the recorded data. Since the SNR directly depends on the number of photons recorded in any particular channel, the spectral width of recording channels must be sufficiently high to achieve good SNR, resulting in the typical use of small numbers of spectral channels.

Previously we developed a biological labeling, image acquisition and image analysis technique that combined both combinatorial labeling and spectral imaging to allow us to distinguish 28 different kinds of objects in a single fluorescence image [15]. Each different kind of object is labeled with a specific combination of fluorophores, giving each object a spectral signature. Here, we couple this technique with an unmixing algorithm that uses information about how the spectral images were acquired and *a priori* knowledge about how the specimens were labeled to greatly increase the number of distinguishable objects.

We record emission spectra at multiple excitation wavelengths and concatenate the spectrally recorded data, effectively using both excitation and emission spectral information in the unmixing problem. This increases the number of observations in the linear system and therefore the number of unique solutions for identifying individual fluorophores. The key feature of our algorithm is the use of a priori knowledge that is generated by labeling each organism with exactly two fluorophores. This knowledge allows us to more robustly solve the unmixing problem and greatly expand the number of identifiable fluorophores.

## Materials and Methods

### Cells and FISH

Escherichia coli K12 (ATCC 10798) cells were grown to an OD_600_ of 1 in Luria Bertani (LB) broth (Beckton Dickinson, Franklin Lakes, NJ, USA) then harvested. Cells were fixed by incubation in 2% paraformaldehyde at room temperature for 1.5 hours. FISH was performed in Eppendorf tubes using a protocol modified from Pernthaler, et al. Cells were labeled with the EUB-338 probe (5’-GCT GCC TCC CGT AGG AGT-3’), which targets a highly conserved region of the 16S rRNA [16]. Separate aliquots of cells were prepared in microcentrifuge tubes and suspended in FISH hybridization buffer. To each of the tubes, either a single or a binary combination of fluorophore-labeled probes were added to give a final probe concentration of 2 μM. After hybridization, cells were washed in wash 1 buffer (0.9M NaCl, 0.02 M Tris (pH 7.5), 0.01% SDS, 20% formamide) for 15 min at 48°C and then in wash 2 buffer (0.9M NaCL, 0.02 M Tris (pH 7.5), 0.01% SDS) for 15 min at 45°C. Cells were pelleted then resuspended in 0.025 M NaCl + 0.02 M Tris (pH 7.5). Five to 125 microliters of each of the cell suspensions from each tube was pipetted into a single tube to give a mixture which then was spotted on an Ultrastick slide (Thermofisher). The slide was placed in a humid chamber for 60 min at room temperature to allow cells to settle and then was rinsed very briefly in ice cold ethyl alcohol and allowed to air dry. The dried specimens were mounted in Vectashield antifade solution (Vector Laboratories).

### Image acquisition

Spectral images were acquired with either a Nikon A1 (Nikon Instruments, Melville, NY, USA) or a Zeiss 710 (Carl Zeiss, Inc., Thornwood, NY, USA) laser scanning confocal microscope each equipped with a 32-channel multianode spectral detector and six laser lines. Images of singly labeled cells were acquired with a 63x/1.4 NA objective lens and images of binary-labeled cells were acquired with a 20x/0.8 NA objective lens. Spectral images were acquired with either 5 nm (Nikon) or 9.7 nm (Zeiss) channel widths, meaning that each of the available 32 anodes on the spectral detector collected light over a 5 or 9.7 nm bandwidth of the visible and near infrared spectrum. Sequential spectral image acquisitions were made in order of descending wavelength of the excitation laser light: 633 nm, 594 nm, 561 nm, 514 nm, 488 nm, and 405 nm. A total of 115 separate excitation/emission combinations were acquired for each field of view. Spectral images were imported as TIFF files into *Mathematica* (Wolfram Research, Inc., Champaign, IL, USA) for unmixing.

### Linear Unmixing

A custom linear unmixing algorithm was written on the Mathematica platform v8. Images were imported into Mathematica as TIFF files. For every pixel or segmented object in an image, unconstrained linear unmixing was first performed using the Mathematica function LeastSquares[], solving for the abundance of all fluorophores. The solution to the least-squares problem was then evaluated. If all fluorophore abundances were positive, the result was accepted. If any of the fluorophore abundances were negative, the result was rejected and a constrained linear unmixing operation was performed using the Mathematica function FindMinimum[], with Method set to "Quadratic Programming." The FindMinimum[] approach to solving the least squares problem invokes a non-negativity constraint since it is not physically possible for a fluorophore to have a negative abundance. However, this iterative nonlinear method is computationally intensive and slow. Therefore the unconstrained LeastSquares[] function was applied at every pixel first.

### Description of the algorithm

Linear unmixing algorithms as implemented for multispectral fluorescence microscope data involve solving a system of linear equations with the general form:

$$\begin{array}{c} y_{1}\\ \vdots \\ y_{m} \end{array}=\left[\begin{array}{ccc} f_{1,1} & \cdots & f_{1,n}\\ \vdots & \ddots & \vdots \\ f_{m,1} & \cdots & f_{m,n} \end{array}\right]\cdot \begin{array}{c} x_{1}\\ \vdots \\ x_{n} \end{array}$$ (1)

For ease of presentation in a text block, Eq 1 may be rewritten as:

y=F⋅x (2)

where ***y*** is a column vector of observed fluorescence intensities in *m* number of different spectral channels, **F** is an *m × n* matrix of known fluorescence spectra consisting of the *m* spectral channels for each of the *n* number of fluorophores used in an experiment, and ***x*** is the computed abundance of each of the *n* fluorophores [17]. A least squares method to minimize the residuals is generally performed to solve for ***x***. In practice, common constraints such as non-negativity and abundance sum-to-one requirements are imposed. Our image analysis pipeline, which is designed to greatly expand the number of distinguishable entities, institutes three operations different from this standard approach: concatenation of multiple excitation images, averaging of pixel intensities over all pixels in a segmented object in the raw spectral image before unmixing, and a binary label constraint on the least squares solution for ***x***.

### Concatenation

**Fig 1:**
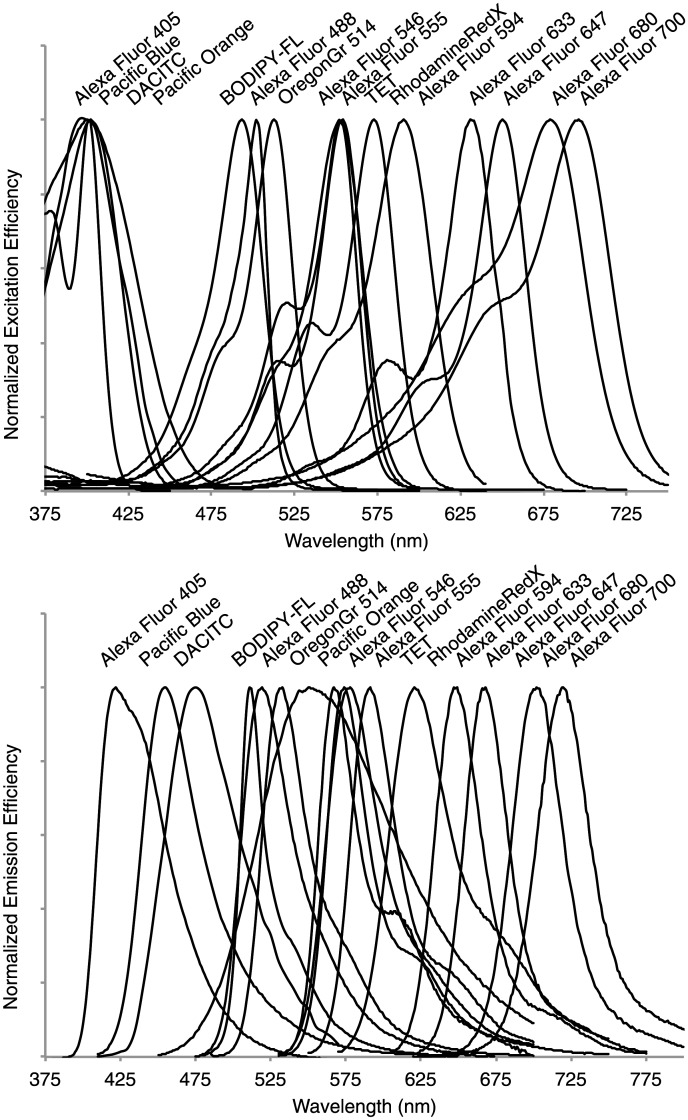
Excitation and emission spectra of 16 commercially available fluorophores. Both excitation (A) and emission (B) spectra of organic fluorophores have characteristic shapes.

In addition to emission spectra with characteristic shapes plotted as emitted intensity against wavelength, fluorophores also have unique excitation spectra, which are just as characteristic for their identification (Fig 1). Our algorithm combines the data from several separate spectral images of the same field of view with each image acquired using excitation light of a different wavelength. With multiple separate excitations, we have a system of linear equations for each excitation, which can be considered together as a single linear system.

Although the shape of emission spectra is generally unaffected by excitation wavelength, the emission intensity of a fluorophore is partly a function of the cross sectional area of the excitation wavelength band and the fluorophore emission curve. In practice, when fluorophores are excited with different wavelengths of light, their emission spectral curves are nearly identical for each excitation wavelength, but their emitted intensities differ. By treating the data from multiple excitations as one system, we effectively use the information available from both excitation and emission spectra in assigning fluorophore abundances at each pixel in a spectral image.

**Fig 2:**
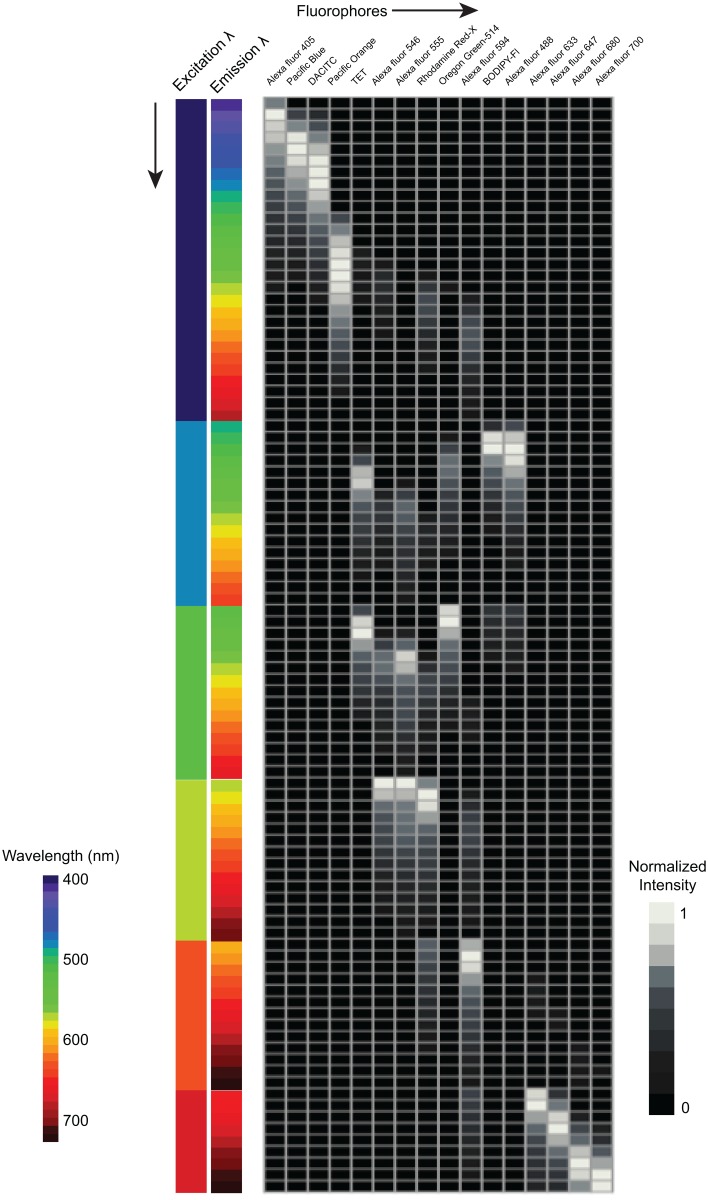
Concatenation of fluorophore emission spectra recorded with multiple excitation wavelengths. Graphic representation of the m × n matrix of fluorophore emission spectra. Each column represents the spectrum of one fluorophore and each row represents the normalized intensities of the fluorophores at a particular excitation / emission wavelength combination. Shade in the boxes of the matrix represents normalized intensity from zero (black) to 1 (white). Intensity values in each column of the matrix were normalized to the maximum value for each fluorophore. The resulting excitation/emission pattern is specific to the combination of excitation wavelengths and excitation energy. In order for this matrix to correctly represent the fluorophore spectra in labeled specimens, the samples must be images using the same excitation wavelength combination and excitation power.

A spectral image is recorded in *m* number of channels where each channel records emission over a certain wavelength range. We typically display the spectrum recorded at any pixel in the image as a plot of intensity vs. wavelength as in Fig 1. However, with *p* excitations, we have *m* × *p* different channels of recorded spectral data, each channel captures a specific emission wavelength band when the sample was excited with light of a particular wavelength. Thus we now have two independent variables: wavelength of excitation and wavelength of emission, along with one dependent variable: intensity. To represent this multidimensional data graphically, we forgo a typical plot of intensity vs. wavelength and rather display the reference spectra as a table since the unmixing operation requires as input the known reference spectra in the form of a mathematical matrix (Fig 2). In this graphic, for each fluorophore the intensity at every emission wavelength band for the shortest wavelength excitation, 405 nm, is plotted at the top of the matrix. Then, added end-to-end is the fluorophore intensity at every emission wavelength for the next longer wavelength excitation, 488 nm. Each successively longer wavelength excitation data set is thus combined end-to-end, or concatenated, to the previous data set, giving a final spectrum that has *m* × *p* data points. After concatenation, each reference spectrum is normalized to its peak intensity such that the signal in the overall highest intensity excitation / emission channel is considered unit intensity and all other channels are some fraction of this unit.

Once the excitation / emission reference spectra matrix was assembled, we used this matrix to unmix concatenated spectral images of a mixture of 16 differently labeled *E*. *coli*. Images were imported into *Mathematica* for concatenation and unmixing. The unmixing algorithm converts the 115-channel spectral image into a 16-channel image in which the intensity at each pixel in every channel represents the abundance of each of the 16 fluorophores used in the experiment.

### Averaging

In order to assign each segmented object its fluorophore identity, the intensity values for every pixel that make up each object may be averaged. This process of averaging pixel intensities may be performed after the images have been unmixed, in which case an average value for all fluorophores used in the experiment would be obtained and the channels with the highest average fluorophore abundance at each object would identify that object's fluorophore composition. Alternatively, the spectrally-recorded image may be segmented and the pixel values may be averaged in the raw spectral image, before unmixing. One advantage to the second approach comes in the form of computational efficiency. In an image of fluorescently labeled bacteria, there may be as few as 100 objects, yet the image might consist of 1024 x 1024 = 1,048,576 pixels. Solving the linear unmixing problem for every cell in the image, rather than every pixel would result in an approximately 10,000-fold savings in computational resource. Furthermore, averaging pixel intensities over all pixels in a segmented feature effectively reduces noise in the recorded spectrum, resulting in more accurate fluorophore assignment after linear unmixing. For segmenting raw spectral images, an algorithmic, histogram-based method was used to determine a threshold intensity. 59 high signal-to-noise spectral channels that spanned the data set were used for thresholding. No pixels were excluded before averaging.

### Binary label constraint

A priori knowledge can enhance spectral analysis. If it is known that every object in an image is labeled with, for example, exactly two fluorophores, we reasoned that a more accurate description of the image would come from an unmixing algorithm that finds the best fit of exactly two fluorophores to each individual object, rather than all of the fluorophores that might be present somewhere in the image.

This binary label constraint can no longer be realized within a linear unmixing framework, but is implemented as follows. From the entire repertoire of fluorophores used in an experiment, for example the 16 fluorophores plotted in Fig 1, and which compose the matrix **F** in Eq 1, all the possible subsets of two fluorophores are identified, giving, in this case, 120 binary subsets. These 120 binary fluorophore subsets represent all the possible label types that would be present in an image of labeled microbes if all the possible binary combinations of 16 fluorophores were used to label the cells. Eq 1 is then rewritten as:

$$\begin{array}{c} y_{1}\\ \vdots \\ y_{m} \end{array}=\left(\begin{array}{c} f_{1,i}\\ \vdots \\ f_{m,i} \end{array}\cdot x_{i}\right)+\left(\begin{array}{c} f_{1,j}\\ \vdots \\ f_{m,j} \end{array}\cdot x_{j}\right)+\begin{array}{c} b_{1}\\ \vdots \\ b_{m} \end{array}$$ (3)

where [*f*_*i*_, *f*_*j*_] ϵ **F**

where the column vector with elements *y*_*1*…*m*_ is once again the observed spectrum at a pixel or segmented object in the spectral image, **F** is the matrix of known fluorophore reference spectra, the vectors *f*_*i*,*1*…*m*_ and *f*_*j*,*1*…*m*_ are two fluorophore spectra from the entire repertoire in **F**, and *x*_*i*_ and *x*_*j*_ are the abundances of each of the fluorophores *i* and *j*, present in the object. Together, *x*_*i*_ and *x*_*j*_ make a 2 by 1 column vector, as *x* in Eq 2 is an *n* by 1 vector with *n* being the total number of fluorophores used in the experiment. The vector with elements *b*_*1*…*m*_ represents background signal in the image. To determine background, a region of interest (ROI) is identified in the image where no cells are present, then, the measured spectrum in all the pixels in the ROI is averaged. Background is assumed to consist mostly of stray light and is further assumed to be the same for all objects in the image. Other sources of noise, e.g., detector noise and photon shot noise, are expected to be different at each pixel and are not treated as a constant term, but contribute to the residuals calculated as the difference between the observed result and the theoretical best fit solution.

**Fig 3:**
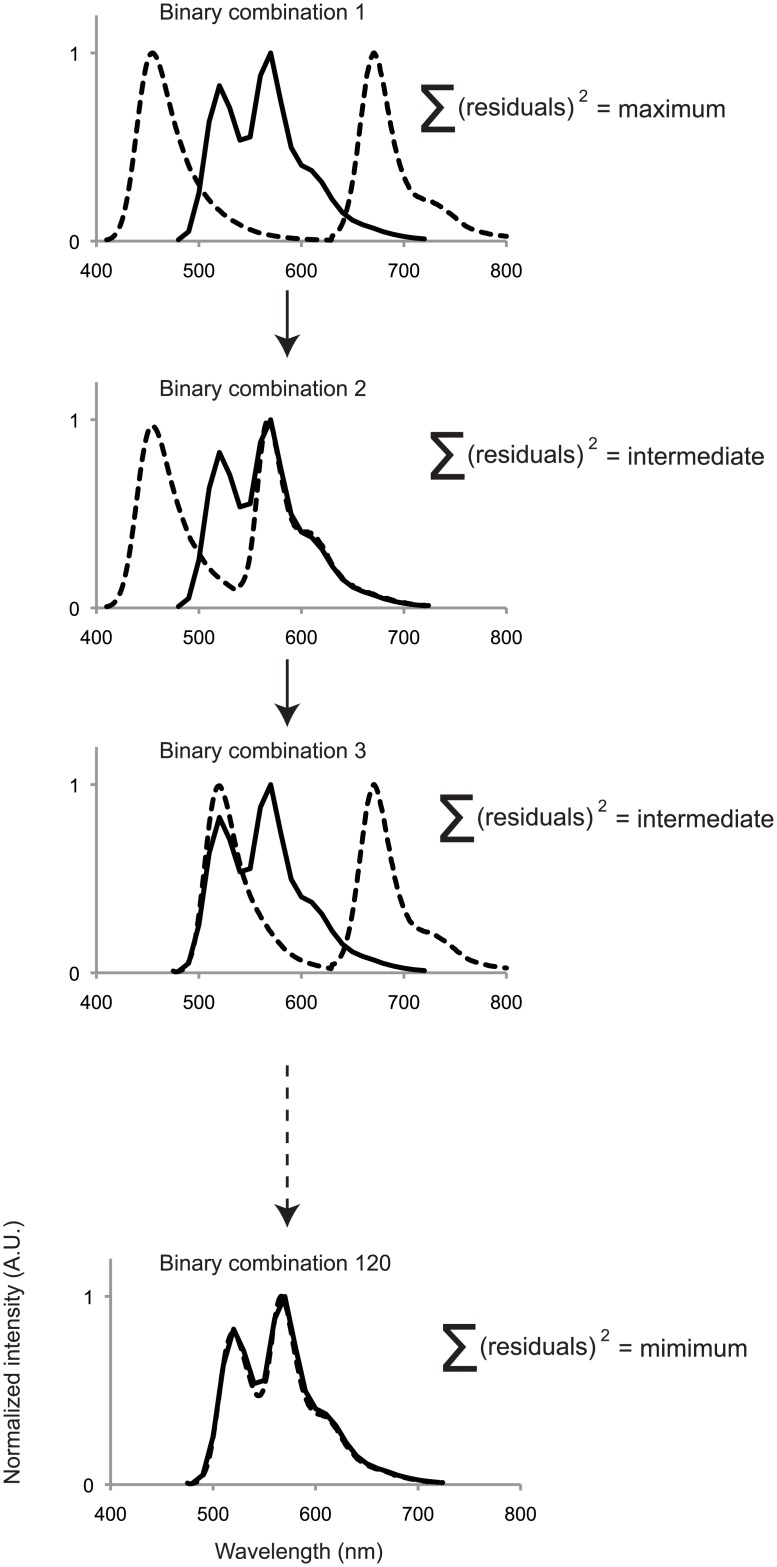
Graphic representation of the binary label constraint on the unmixing solution. In this in-silico experiment, we depict an observed emission spectrum of an object labeled with AlexaFluor 488 and AlexaFluor 555 (solid line). Dotted lines represent possible binary combinations of reference spectra. While the graphic only shows 4 different binary combinations, the algorithm solves the unmixing operation for 120 combinations, each time finding the best fit of the binary spectrum to the observed spectrum. Once all 120 solutions are found, the solution with the overall lowest sum of squared residuals is identified and that combination is chosen as the best solution.

In Eq 3, *x*_*i*_ and *x*_*j*_ are solved-for independently by linear regression for each unique subset, {*i*,*j*} of fluorophores in the repertoire. For example, if there are 16 fluorophores and 120 potential label-types in an image, then Eq 3 is solved, in series, 120 times, each time with a unique pair of *f*_*i*_ and *f*_*j*_ reference spectra from the pool of 16 spectra in **F**. Thus, each object in the image will have 120 solutions, each solution consisting of a pair of fluorophore abundance values and a sum of squared residuals, together representing one of 120 best fits to the recorded spectrum in that object. In the final algorithm step, the pair of fluorophores that produced the smallest sum of squared residuals (Fig 3) is picked for representing the object.

## Results

**Fig 4:**
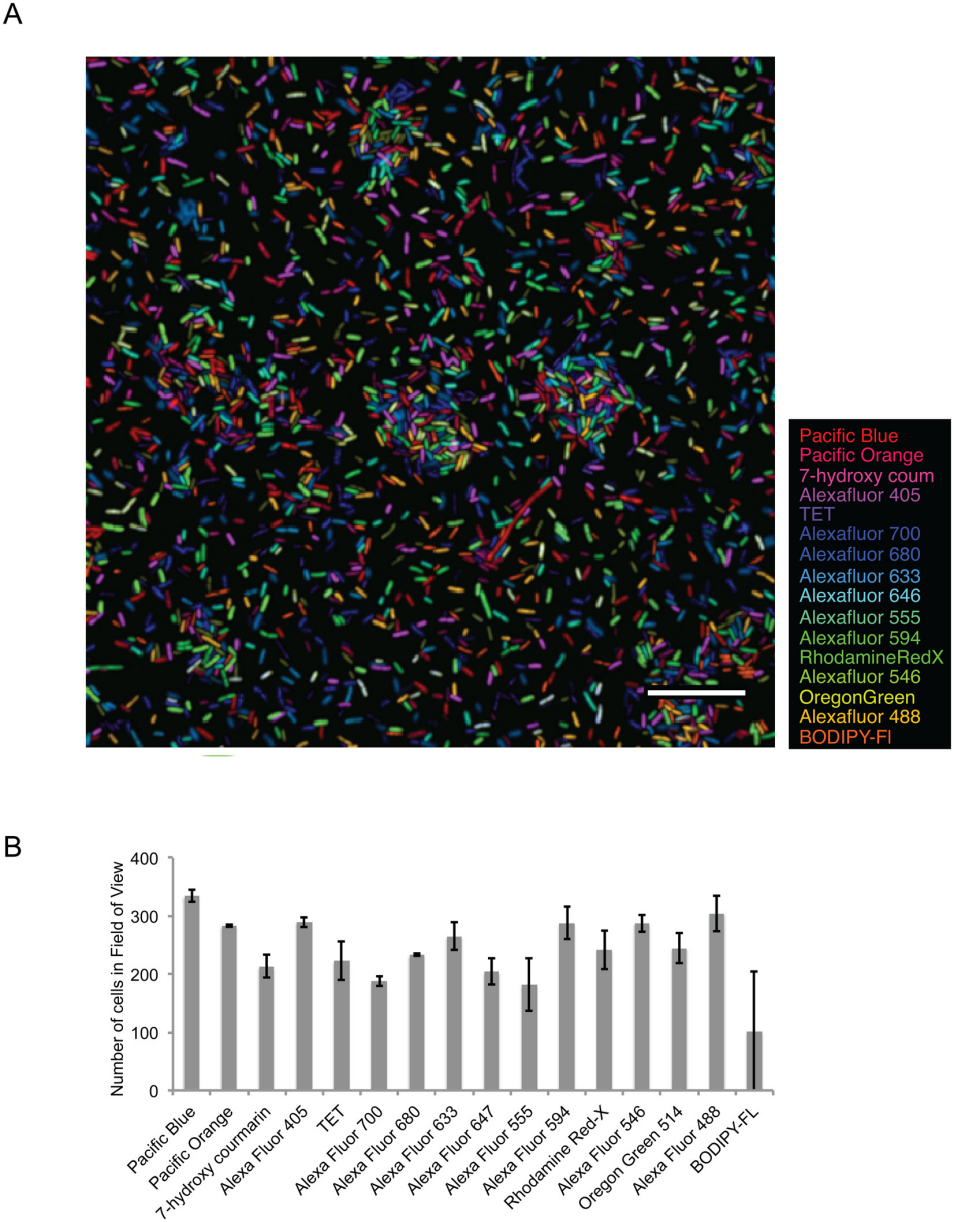
Mixture of 16 differently labeled populations of E. coli **A**. Image of the *E*. *coli* mixture after standard linear unmixing using an algorithm that concatenates spectral data from multiple spectral images of a single field of view. Bar = 20 μm. Present in the center of the image is an abnormally long E. coli cell, which are sometimes present in our laboratory culture. **B**. Quantification of the 16 different label-types. Equal volumes of each label type were combined to create the mixture. All label-types are present in the image at approximately equal concentration as expected. Bars represent mean values from three fields of view of the same mixture and error bars represent standard deviation.

We evaluated the utility of concatenating spectral images acquired at different excitation wavelengths by using multiple populations of *E*. *coli* as test objects. 16 aliquots of *E*. *coli* were labeled in a fluorescence in situ hybridization (FISH) experiment with different versions of the same oligonucleotide probe, the EUB-338 probe [16], each version conjugated to a different fluorophore. The labeled cells were then combined into a mixture at equal concentration and imaged. Images of the mixture of 16 differently labeled *E*. *coli* were acquired so that at each excitation wavelength, some pixels in at least one spectral image channel were close to saturation, such that the full dynamic range of the detector was utilized. Once the optimal image acquisition settings were determined empirically for the mixture, spectral images of pure populations of each of the 16 differently labeled *E*. *coli* were acquired for determining reference spectra, the components of **F** in Eq 2. Presented in Fig 4A is a color merge image in which each of the fluorophore channels was pseudo-colored one of sixteen different colors. After image segmentation and quantitative analysis, all sixteen differently labeled *E*. *coli* were identified in the image and in approximately equal proportion as expected (Fig 4B). The mean number of cells labeled with each fluorophore was 243 cells and all sixteen populations were within one standard deviation (63 cells) of the mean except the BODIPY-FL population.

**Fig 5:**
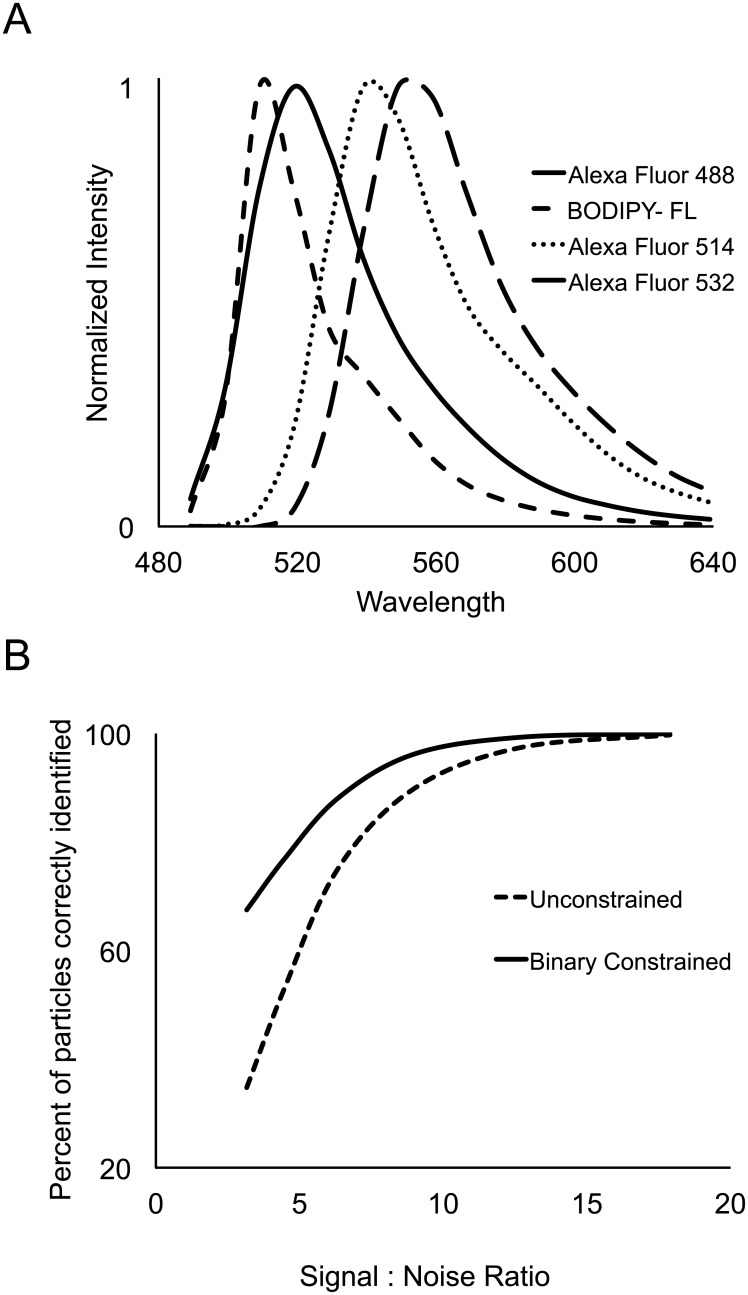
Computer model to test binary label constraint. **A**. Normalized emission spectra of four fluorophores that are well excited by 488 nm laser light. **B**. Computer model data. Model particle spectra were created in *Mathematica* as a binary combination of Alexa Fluor 488 and BODIPY-FL, then unmixed against the spectra of all four fluorophores plotted in A. Results of the unmixing are plotted as the percent of particles that were correctly identified in their binary label composition as a function of signal-to-noise ratio in the modeled spectra either by standard unmixing or with a binary label constraint.

We tested the efficacy of our combinatorial constraint with a computer model. In *Mathematica*, we generated sets of particle spectra, each set modeled as a binary combination of Alexa Fluor 488 and BODIPY-FL with different signal-to-noise ratios. To specifically test the effect of the binary constraint, independent of spectral concatenation, we modeled the data as if it had been acquired with a single excitation at 488 nm collected in a total of 16 channels, each channel corresponded to an emission bandwidth of 10 nm. The spectra were modeled with Poisson distributed shot noise and were unmixed against four possible fluorophores as shown in Fig 5A. Model spectra were unmixed either with or without the binary constraint. Results for a 1:1 mixture of Alexa Fluor 488 and BODIPY-FL are presented in Fig 5B; similar results were obtained for other combinations of fluorophores with overlapping emission spectra. The constrained and unconstrained algorithms were evaluated by asking what percent of modeled particle spectra were correctly identified in their binary fluorophore combinations. At all signal-to-noise ratios tested, and especially at low signal-to-noise ratios, the binary constrained algorithm outperformed the unconstrained algorithm.

### Biological proof of principle

We next sought to establish that microbes labeled with 120 different binary combinations of 16 fluorophores with highly overlapping emission spectra could be distinguished in spectrally acquired microscope images after application of our binary constrained unmixing algorithm. *E*. *coli* were again used as test objects. Sixteen versions of the EUB338 FISH probe were used to label separate aliquots of *E*. *coli* cells such that 120 populations of microbes were created, each population labeled with a unique binary combination of probes, referred to as a label type. After FISH, the separately labeled *E*. *coli* populations were combined to create a mixture of the 120 different label types in such a way that each of the label types would be present in the mixture at different concentrations, ranging linearly from 0.12% to 1.5% of the total.

Specimens were imaged with a laser scanning confocal microscope equipped with a spectral detector. Separate spectral image acquisitions were made of each field of view of labeled *E*. *coli*, with each image acquired using a different laser line available on the microscope system for fluorophore excitation. The spectral images for each field of view were concatenated into a single spectral image data set as described previously.

**Fig 6:**
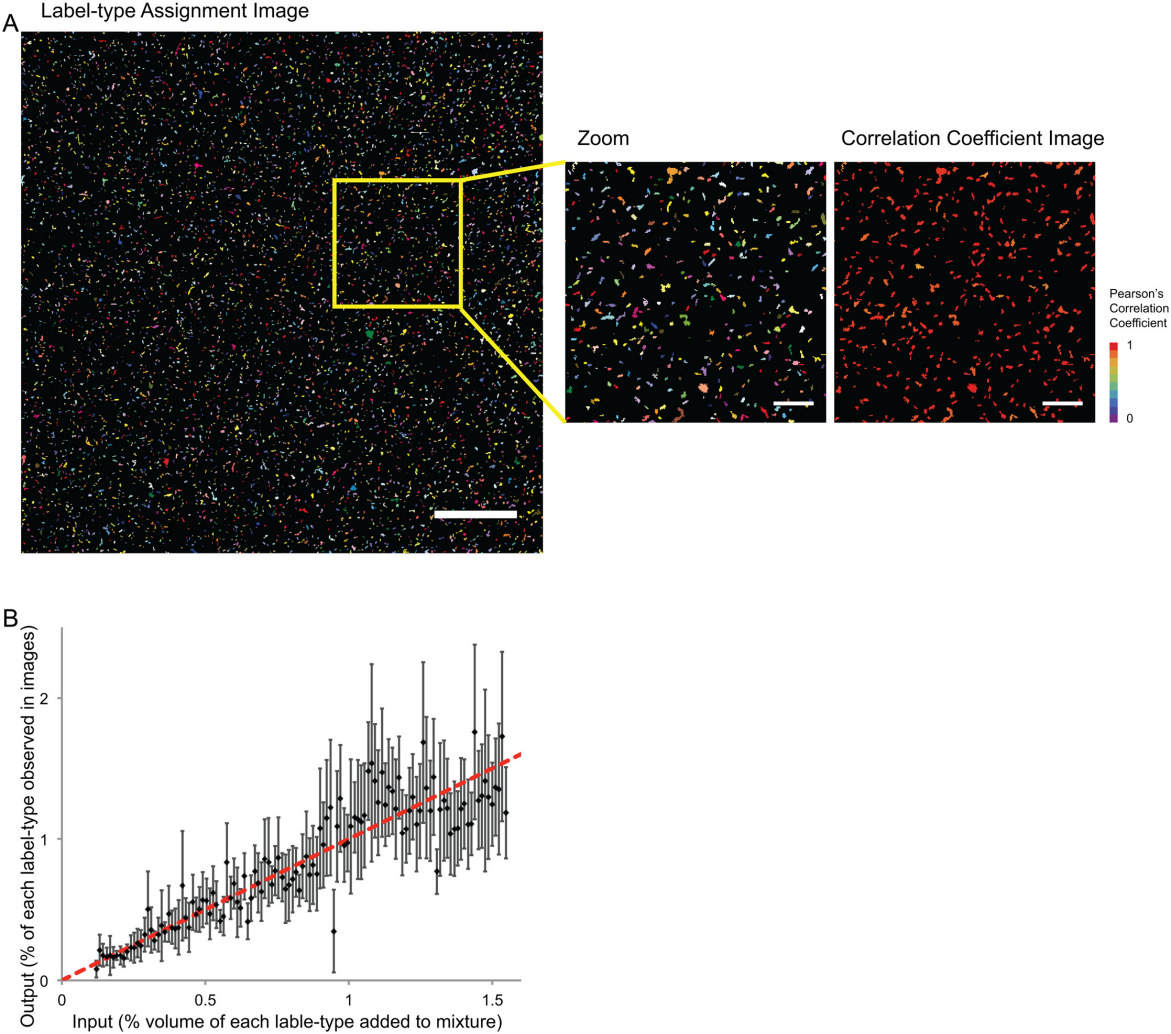
Proof-of-principle experiment with 120 differently labeled E. coli **A**. Unmixed spectral image of an artificial mixture of 120 differently labeled E. coli, each of the 120 label types being a binary combination of two fluorophores from the repertoire of 16 in Fig 1. Cells were segmented from background in raw spectral images, intensity measurements were averaged over all pixels within each cell, then the averaged object spectra were unmixed using the binary-constrained algorithm described in the text. Each binary label-type is represented as a unique color. The image was acquired with a low magnification, high numerical aperture objective lens (20 X/0.8 NA). Bar = 100 μm. Inset shows a magnified region of interest from the unmixed image and a graphical representation of confidence in label assignment of each particle in the image. Confidence level is reported as the Pearson’s correlation coefficient between observed spectrum and computed model spectrum from the best-fit unmixing solution. Bar = 25 μm. **B**. Output from unmixed images, measured as the percent of each label-type of cell detected in the mixture is plotted against input into the mixture, measured as percent volume of each label-type added to make the mixture. Dashed, red line indicates the expected Input-to-Output relationship, (slope = 1). Error bars represent standard deviation in the percent output values from 8 different fields of view of the same artificial mixture.

Spectral images were segmented into particles as described in Methods. Concatenated, segmented images were then fed into our combinatorially-constrained algorithm for unmixing. The output from the unmixing operation is a single binary image in which particles represent microbial cells in the specimen (Fig 6). Each feature is pseudo-colored in one of 120 different colors according to its label type. Finally, we compared input into the mixture with output from the unmixed image data and found that the Pearson’s correlation coefficient between the two measures was 0.91. Input for each label-type was the volume of cell suspension added to make the mixture. All label-types were prepared such that an equal number of cells was pipetted into each tube. However, after multiple rounds of pelleting and washing some pipetting error is expected.

### Error Analysis

Our unmixing algorithm finds the overall best-fit solution to the fluorophore identity and abundance problem. However, the best-fit solution may incorrectly identify one or both fluorophores, especially when intensities recorded for a given particle have low signal to noise ratios. As a measure of confidence in the assigned particle identity, we calculate the Pearson's correlation coefficient for each segmented particle (Fig 6A) between the observed spectrum for that particle and the computed spectrum for the best binary fit.

**Fig 7:**
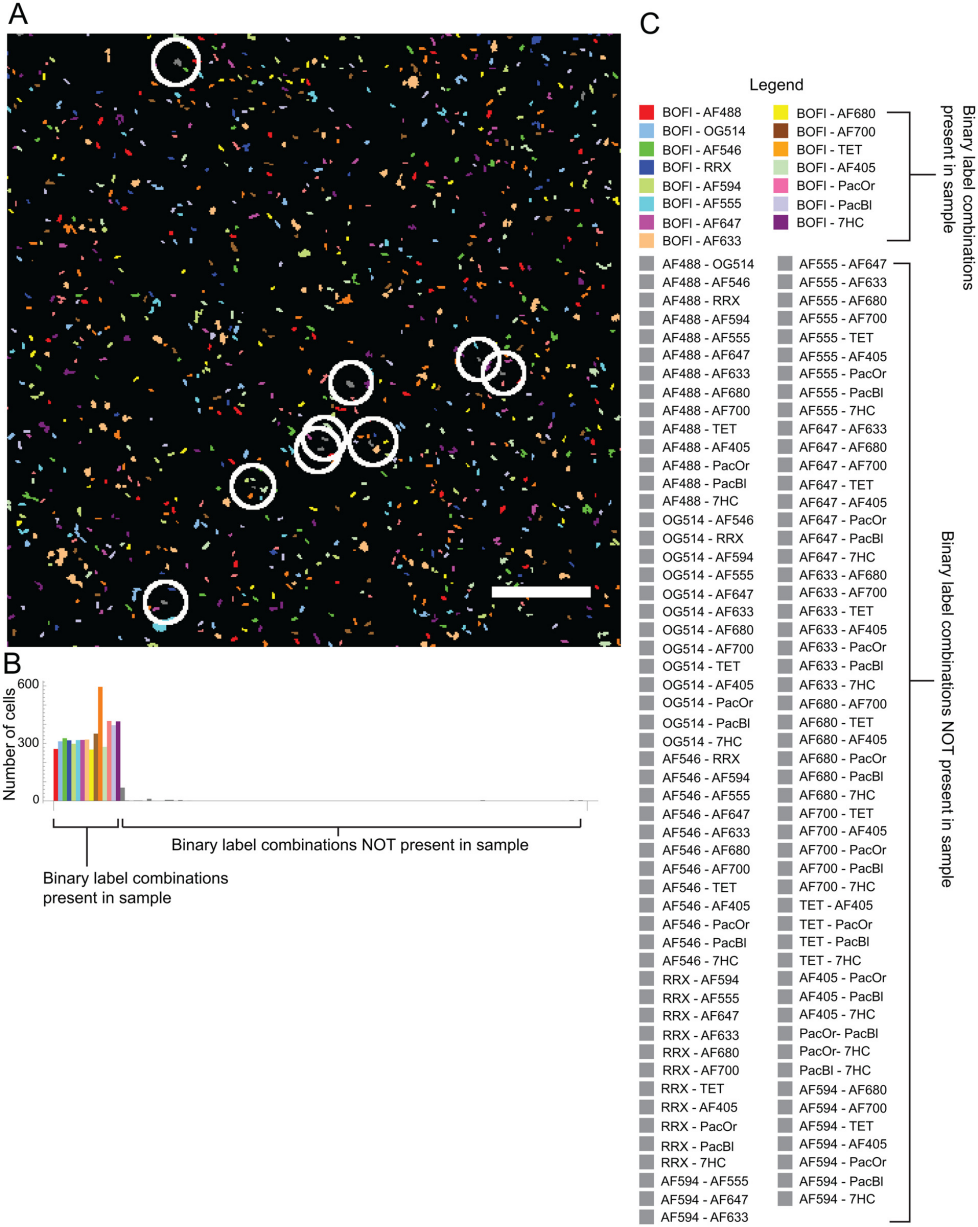
Test of accuracy of binary label identification. **A**. Unmixed spectral image of an artificial mixture of 15 differently labeled E. coli, each of the 15 label types being a binary combination of BODIPY-Fl with one of the other fluorophores from the repertoire of 16 in Fig 1. Cells were segmented from background in raw spectral images, intensity measurements were averaged over all pixels within each cell, then the averaged object spectra were unmixed using the binary-constrained algorithm described in the text, such that all 120 binary label types were possible solutions. Each of the 15 binary label-types known to be present in the sample is represented as a unique color, all 105 label types known not to be present in the mixture are colored gray. Circles denote all of the gray cells in the image. The image was acquired with a low magnification, high numerical aperture objective lens (20 X/0.8 NA). Bar = 100 μm. **B**. Quantification of label types. The number of cells of each of the 120 possible label types in the image in A were counted. Colored bars represent label types known to be present in the sample, gray bars represent label types known not to be present in the sample. **C**. Legend for (**A)** and (**B)**. BOFl = BODIPY Fl, AF488 = Alexa Flour 488, OG514 = Oregon Green 514, AF546 = Alexa fluor 546, RRX = Rhodamine Red-X, AF594 = Alexa fluor 594, AF555 = Alexa fluor 555, AF647 = Alexa fluor 647, AF633 = Alexa fluor 633, AF680 = Alexa fluor 680, AF700 = Alexa flor 700, TET = Tetramethyl rhodamine, AF405 = Alexa fluor 405, PacOr = Pacific Orange, PacBl = Pacific Blue, 7HC = 7-Hydroxy coumarin.

To test the accuracy of the binary label assignment, we prepared an artificial mixture of binary-labeled microbes that contained only a subset of the 120 label types, namely all 15 binary label types that included BODIPY Fl, in equal proportion. We spotted the mixtures on a slide, and imaged the mixture using the same image acquisition settings as for the mixtures of all 120 binary labeled microbes. We then unmixed the images using the binary constrained unmixing algorithm that allowed all 120 possible label types to be present (Fig 7A and 7C).

We counted the number of each of the label types that should be present in the image, i.e., the 15 binary label types that contained BODIPY Fl as one of the labels (Fig 7B and 7C). We further counted the number of impossible label types, i.e, the 105 binary labels that did not contain BODIPY Fl and were known to not be in the mixture. We found that of the 5313 cells in the image, 97.9% were identified as a label type containing BODIPY Fl and 2.1% were identified as an impossible label type (Table 1). We further prepared similar mixtures containing all the binary label types containing either Alexa fluor 546, tetramethyl rhodamine or Alexa fluor 647 and found that the fraction of impossible label types identified in each mixture was acceptably low,—2.7%, 0.3% and 0.1% respectively (Table 1).

**Table 1:** Accuracy test of binary label identification.

Binary label repertoire	Total number of cells in image	Percent possible label types	Percent impossible label types
BODIPY- Fl	5313	97.9%	2.1%
Alexa fluor 546	5129	97.3%	2.7%
Tetramethyl rhodamine	2978	99.7%	0.3%
Alexa fluor 647	3134	99.9%	0.1%

## Discussion

We have developed a spectral imaging analysis pipeline to distinguish 120 differently labeled microbes in a single acquired image. Our algorithm uses *a priori* knowledge about images of combinatorially labeled specimens and includes the following three features: 1. Concatenation of spectral images acquired in series with multiple wavelength excitation; 2. averaging of spectral intensities over all the pixels within segmented particles before unmixing; and 3. constraining the unmixing solution to the best fit of exactly two fluorophores for every particle in the image. Some of the features of our algorithm should be widely applicable to many applications of biological fluorescence imaging i.e., concatenation and particle averaging, while the binary unmixing constraint is particularly suited to combinatorial labeling of microbes as presented here.

Sequential excitation with 6 different wavelengths and subsequent concatenation of spectra allowed us to use information about both the excitation an demission spectra of fluorophores for their identification. Still, because a linear unmixing operation is solved as a system of linear equations using the method of least squares, the number of observations (spectral channels) must be equal to or greater than the number of unknowns (fluorophores) used. Here we have demonstrated that by adding steps to the unmixing algorithm that exploit a priori knowledge about the specimen, i.e. that each microbe is labeled with exactly two fluorophores, we can distinguish more label combinations than we have spectral channels. In practice, exciting a sample at multiple wavelengths in series increases the overall time needed to acquire an image. For example, a single excitation wavelength image (1024 pixels squared) is typically acquired in ~6.7 s with a pixel dwell time of 6.4 μs. With six separate excitations, the total time to acquire the image is 40s. Although practical for fixed samples, this is incompatible with live-cell imaging. Nonetheless, single excitation spectral imaging can be used for dynamic samples with the following caveat: as the number of separate excitation wavelengths is decreased, so is the ability to distinguish multiple fluorophores.

Implicit in our novel algorithm is the assumption that each segmented feature in the recorded image represents a single combinatorially labeled object in the image. This assumption is valid for well-dispersed cells as in Fig 6, but may not hold when two objects are very close to each other, especially in the axial dimension, and are then segmented together during image processing as one feature. When objects are expected to overlap in the recorded image, specimens may be labeled with single fluorophores, rather than combinations. An unmixing algorithm that retains the concatenation of images recorded with multiple excitation wavelengths but that disposes of the combinatorial constraint could then be used. We have demonstrated an ability to label and identify 16 different kinds of microbial cells in a single specimen with this type of approach. We expect future improvements in segmentation algorithms, especially segmentation in the axial dimension, to extend the utility of the combinatorially constrained algorithm.

We have demonstrated an extraordinary ability to distinguish fluorophores with highly overlapping emission spectra by combining both excitation and emission spectral data in a single linear unmixing algorithm. In order to maximize the percentage of correctly identified objects, reference spectra must accurately represent the fluorophore spectra in the acquired image. To ensure representative reference spectra, they should be acquired from pure populations of similar, singly labeled objects, and with exactly the same instrument settings, e.g., laser power, detector gain, exposure time, as used for acquiring the specimen image. Furthermore, fluorophore bleaching, which can impart artifacts in the excitation / emission profile of a fluorophore must be kept to a minimum. To minimize bleaching, images need to be acquired with the longest wavelength excitation first, followed by the second longest and so on, down to shortest wavelength. Additionally, reference spectra recorded with pure populations need also be recorded with the same sequence, from longest to shortest excitation wavelength, as used for the biological specimens of interest. This will minimize the effect of bleaching artifacts because both the reference spectra and the unknown samples bleach with similar dynamics. In the future, our excitation emission concatenation protocol could be improved with appropriate mathematical corrections for fluorophore bleaching.

In summary, the ability to label and distinguish tens to hundreds of objects in a single fluorescence image as presented here will offer new avenues for discovery and study of complex biological organization. With the concurrent development of appropriate imaging informatics analysis procedures this new ability could allow the systems-level analysis of many complex biological structures from microbial biofilms to eukaryotic cells and tissues.
